# Release from natural enemies mitigates inbreeding depression in native and invasive *Silene latifolia* populations

**DOI:** 10.1002/ece3.4990

**Published:** 2019-02-18

**Authors:** Karin Schrieber, Sabrina Wolf, Catherina Wypior, Diana Höhlig, Stephen R. Keller, Isabell Hensen, Susanne Lachmuth

**Affiliations:** ^1^ Department of Chemical Ecology, Faculty of Biology Bielefeld University Bielefeld Germany; ^2^ Geobotany & Botanical Garden, Institute of Biology Martin‐Luther‐University Halle‐Wittenberg Halle (Saale) Germany; ^3^ Department of Plant Biology University of Vermont Burlington Vermont; ^4^ German Centre for Integrative Biodiversity Research (iDiv) Halle‐Jena‐Leipzig Leipzig Germany

**Keywords:** biological invasion, plant defense, evolution, genetic paradox, herbivory, inbreeding, purging, *Silene latifolia*

## Abstract

Inbreeding and enemy infestation are common in plants and can synergistically reduce their performance. This inbreeding ×environment (I × E) interaction may be of particular importance for the success of plant invasions if introduced populations experience a release from attack by natural enemies relative to their native conspecifics. Here, we investigate whether inbreeding affects plant infestation damage, whether inbreeding depression in growth and reproduction is mitigated by enemy release, and whether this effect is more pronounced in invasive than native plant populations. We used the invader *Silene latifolia* and its natural enemies as a study system. We performed two generations of experimental out‐ and inbreeding within eight native (European) and eight invasive (North American) populations under controlled conditions using field‐collected seeds. Subsequently, we exposed the offspring to an enemy exclusion and inclusion treatment in a common garden in the species’ native range to assess the interactive effects of population origin (range), breeding treatment, and enemy treatment on infestation damage, growth, and reproduction. Inbreeding increased flower and leaf infestation damage in plants from both ranges, but had opposing effects on fruit damage in native versus invasive plants. Inbreeding significantly reduced plant fitness; whereby, inbreeding depression in fruit number was higher in enemy inclusions than exclusions. This effect was equally pronounced in populations from both distribution ranges. Moreover, the magnitude of inbreeding depression in fruit number was lower in invasive than native populations. These results support that inbreeding has the potential to reduce plant defenses in *S. latifolia*, which magnifies inbreeding depression in the presence of enemies. However, future studies are necessary to further explore whether enemy release in the invaded habitat has actually decreased inbreeding depression and thus facilitated the persistence of inbred founder populations and invasion success.

## 1 INTRODUCTION

Understanding the forces that promote or prevent species range expansions remains a challenging goal in ecology (Barrett, 2015). During invasion of a new range, populations can be simultaneously exposed to increased inbreeding following founder effects (Schrieber & Lachmuth, 2017) and to substantial alterations in the biotic and abiotic environment (Catford, Jansson, & Nilsson, 2009). Inbreeding and environmental change are known to interact in affecting individual fitness (Fox & Reed, 2011; Kristensen, Pedersen, Vermeulen, & Loeschcke, 2010), population growth (Liao & Reed, 2009), and colonization abilities (Hufbauer, Rutschmann, Serrate, Vermeil de Conchard, & Facon, 2013). Such inbreeding × environment (I × E) interactions are increasingly perceived as potential determinants of species ranges and their dynamics under global change (Colautti, Alexander, Dlugosch, Keller, & Sultan, 2017; Leimu, Vergeer, Angeloni, & Ouborg, 2010; Reed, Fox, Enders, & Kristensen, 2012; Schrieber & Lachmuth, 2017). Nevertheless, empirical studies on the environmental dependency of inbreeding effects in the context of invasions are scarce despite their potential relevance for the prediction and management of invasive species.

Inbreeding can reduce individual fitness in the offspring generation. This inbreeding depression arises from the enhanced phenotypic expression of deleterious recessive mutations (dominance) and the reduced expression of heterozygote advantage (overdominance) following increases in homozygosity (Charlesworth & Willis, 2009). Besides developmental processes and fundamental housekeeping functions, inbreeding can also disrupt responses to insect herbivory in flowering plants (reviewed in Carr & Eubanks, 2014). As compared to outbred plants, inbreds may exhibit a lower expression of genes involved in the induction of defense compounds (Kariyat, Mena‐Alí et al., 2012; Portman, Kariyat, Johnston, Stephenson, & Marden, 2015), release reduced amounts of phytohormones essential for defense signaling (Campbell, Halitschke, Thaler, & Kessler, 2014), produce lower amounts of metabolites mediating direct or indirect defense (Campbell, Thaler, & Kessler, 2013; Kariyat, Mauck et al., 2013; Kariyat, Mauck, Moraes, Stephenson, & Mescher, 2012), or exhibit reduced structural defenses (Kariyat, Balogh et al., 2013). This can increase feeding damage on inbred plants, which in turn magnifies inbreeding depression in the presence of herbivores (Campbell et al., 2013; Carr & Eubanks, 2002) and causes negative feedback on plant population growth (Steets, Knight, & Ashman, 2007). The interactive effects of inbreeding and herbivory on fitness thus contribute substantially to the micro‐ and macroevolution of plant reproductive systems and defense strategies (Carr & Eubanks, 2014; Johnson, Campbell, & Barrett, 2015).

In addition, inbreeding × herbivory interactions may provide a hitherto underappreciated explanation for invasion success in the face of repeated founder effects. During range expansion, plants often escape from their coevolved herbivores and pathogens, while host switching by native enemies in the introduced range mostly occurs with some time delay (Colautti, Ricciardi, Grigorovich, & MacIsaac, 2004; Dietz & Edwards, 2006; Mitchell, Blumenthal, Jarošík, Puckett, & Pyšek, 2010; Mitchell & Power, 2003). Hence, enemy attack is specifically reduced during initial introduction and towards the leading edge of range expansion, where inbreeding rates in plant populations are highest (Schrieber & Lachmuth, 2017). Enemy release may mitigate inbreeding depression in these founding plant populations, increase their persistence, and thus foster plant invasion success. Studies quantifying inbreeding depression in native and introduced plant populations in the presence versus absence of their native natural enemies are a first step to test this assumption.

Such studies can also yield information on how genetic differentiation among plant populations impacts the outcome of I × E interactions, which may help to explain reported inconsistency in their effects on plant fitness (Fox & Reed, 2011; Sandner & Matthies, 2016). During invasions, plant species often evolve changes in performance traits (e.g., increased growth, reproductive output, and competitive ability) and chemical traits (reduced defense against specialists, increased defense against generalists, changes in inducibility and constitutive amounts of defense compounds, and increased allelopathy) (Agrawal et al., 2015; Joshi & Vrieling, 2005; Uesugi & Kessler, 2013). This divergence can arise either from adaptive responses to changes in the selective regime for natural enemies and various other environmental factors (Atwood & Meyerson, 2011; Colautti & Barrett, 2013) or from genetic drift (Keller & Taylor, 2008; Lachmuth, Durka, & Schurr, 2011). Both adaptive and nonadaptive genetic differentiation may likely also have altered the genetic architecture underlying inbreeding depression and its dependency on herbivory, specifically through differences in the accumulation and purging (i.e., negative selection of deleterious recessive mutations in inbred populations) of genetic load in defense‐related traits under past population bottlenecks (Schrieber & Lachmuth, 2017): Lower herbivory pressure in invading plant populations may have lead to the accumulation of genetic load in defense traits, whereas high herbivore pressure in the native range may have lead to purging. If such range‐dependent purging occurred under past population bottlenecks, this may magnify the effects of recent inbreeding × herbivory interactions on plant fitness in invasive relative to native populations.

Here, we investigate the combined effects of inbreeding and enemy infestation on the performance of native and invasive populations of *Silene latifolia* Poir. (Caryophyllaceae). During the invasive expansion from Eurasia to North America, the plant species experienced events conducive to the expression of I × E interactions: Introduced plants escaped their natural enemies (Wolfe, 2002) and experienced severe population bottlenecks (Keller, Gilbert, Fields, & Taylor, 2012; Taylor & Keller, 2007) as well as high inbreeding levels in founder populations (Fields & Taylor, 2014; Richards, 2000). Moreover, Schrieber, Schweiger, Kröner, and Müller (2018) demonstrated that inbreeding diminishes metabolic responses to herbivory in populations from both distribution ranges. Finally, invasive populations evolved differences in enemy susceptibility and performance (Blair & Wolfe, 2004; Keller, Sowell, Neiman, Wolfe, & Taylor, 2009; Schrieber et al., 2017; Wolfe, Elzinga, & Biere, 2004), making *S. latifolia* ideally suited for examining the impact of genetic differentiation on the outcomes of I × E interactions. We conducted experimental in‐ and outbreeding within native and invasive *S. latifolia* populations, exposed the offspring to the absence and presence of native natural enemies, and measured traits related to infestation damage (inverse measure of defense), growth, and reproduction to address the following hypotheses: (a) Inbred plants incur higher infestation damage than outbreds. (b) Plant growth and reproduction are lower in inbreds than outbreds (inbreeding depression) and reduced in the presence as compared to the absence of natural enemies. (c) Inbreeding depression in growth and reproduction is stronger in the presence of natural enemies than in their absence (I × E interaction). (d) The effects of inbreeding on infestation damage are stronger in invasive than native plants, which magnifies I × E interaction effects on growth and reproduction in invasive populations.

## 2 MATERIALS AND METHODS

### 2.1 Study system

*Silene latifolia* is a short‐lived perennial herb mainly distributed across ruderal habitats. The plant is dioecious and produces sexually dimorphic flowers pollinated by insects. Females develop large numbers of capsules containing several hundred seeds, which lack a specific dispersal syndrome and are thus mainly dispersed passively and by human activities. Limited seed dispersal and restricted pollen transfer among neighboring plants can lead to restricted gene flow and the formation of kin‐structured patches within populations (McCauley, 1994, 1997). These characteristics have been shown to result in high levels of biparental inbreeding in small, isolated, or recently founded *S. latifolia* populations (Fields & Taylor, 2014; Richards, 2000).

In its native range (Eurasia), *S. latifolia* is attacked by three specialist enemies: *Hadena bicruris*Hufn. (Noctuidae)—a noctuid moth that is a specialist pollinator (adult) and a seed predator (larva) at the same time; *Microbotryum violaceum* (Pers.) G. Deml & Oberw. (Mycrobotryaceae)—a systemic sterilizing fungus; and *Brachycaudus lychnidis* L. (Aphididae)—an aphid that causes flowers to abort due to phloem feeding (Wolfe, 2002). Moreover, native populations are attacked by various leaf‐ and flower‐feeding generalist herbivores, including slugs (mainly *Arion lusitanicus* Mabille (Arionidae)), beetles, thrips, caterpillars (often *Mamestra brassicae* L. (Noctuidae)), and leaf miners as well as by several generalist rust and mildew fungi (Schrieber et al., 2017). In the invaded range (North America), *H. bicruris*is completely absent (Wolfe, 2002), the occurrence of *M. violaceum* is locally restricted to a small region in Virginia (Antonovics, Hood, Thrall, Abrams, & Duthie, 2003), and the abundance of aphids as well as leaf‐ and flower‐feeding generalists is very low relative to the native range (Wolfe, 2002). As a result of adaptive responses to changes in the selective regime concerning enemy attack and climate as well as of genetic drift effects, invasive *S. latifolia* populations exhibit higher growth, reproduction, and susceptibility to enemy infestation than native populations (Blair & Wolfe, 2004; Keller et al., 2009; Schrieber et al., 2017; Wolfe et al., 2004). A trade‐off between growth/reproduction and enemy susceptibility was not detected in this species (Schrieber et al., 2017).

### 2.2 Field sampling and experimental setup

We collected open‐pollinated seeds from eight native and eight invasive *S. latifolia* populations (Supporting Information Figure S1, Table S2). Sampling in the native range focused on regions thought to be the source of introduced populations (broadly, eastern and western Europe), while sampling in the invasive range comprised the geographic regions of initial introduction and early expansion (eastern North America), as identified by Taylor and Keller (2007) and Keller et al. (2012). Within each population, we sampled one capsule (maternal family) from each of five different female plants that were equally distributed over the population area and spatially separated from each other as far as possible (min. 6 m for one female pair in smallest population and ≥10 m for all remaining pairs). Using these field‐collected families, we conducted two generations of experimental inbreeding and outbreeding within all native and invasive populations under controlled greenhouse conditions. The offspring were exposed to the absence and presence of natural enemies in a common garden in the species’ native range. Data for the outbred plants from this experiment have previously been used to investigate adaptive and nonadaptive differentiation in growth, reproduction, and enemy susceptibility between the native and invaded range (Schrieber et al., 2017).

### 2.3 Experimental inbreeding and outbreeding

For the parental generation, we germinated ten seeds from each of the five field‐collected families in 0.8 mM gibberellic acid in a germination chamber (16‐hr light at 25°C, 8‐hr dark at 13°C). After 6 days, the seedlings were planted into pots and transferred to the greenhouse (16‐hr light at 25°C, 8‐hr dark at 13°C) where they received weekly fertilization (Kamasol Brilliant Rot, Compo Expert, Münster, GE). After 7 weeks, we randomly chose one male and one female plant per family for the crossings. Each female received pollen from a sib male belonging to the same family (inbreeding), and pollen from a male belonging to a different family within the same population (outbreeding) at distinct flowers (Supporting Information Figure S3). The crossing of the parental generation resulted in 160 population (N = 16) × family (N = 5) × breeding treatment (N = 2) combinations. For the second generation, we randomly chose one capsule per combination and propagated the F1 plants from its seeds as described for the parental generation. Female inbred offspring received pollen from an inbred male from the same family, while female outbred offspring received pollen from an outbred male from a different family with respect to the relationships created in the first generation (Supporting Information Figure S3). For our breeding design, we decided against an independent pairing of partners or reciprocal crosses over two generations, since these approaches create bias either because they yield many more inbred than outbred lines (independent pairing) or because they do not use the same initial (P generation) gene pool for inbreeding and outbreeding, as more field‐sampled plants are involved in creating the outbred lines than in creating the inbred lines (reciprocal crossing).

We lost seven of the 160 population × family × breeding treatment combinations due to lack of germination, high mortality, lack of flowering, or production of sterile flowers in both inbred and outbred families during the propagation of the F1 generation. Consequently, we obtained a total of 153 population × family × breeding treatment combinations for the F2‐generation, which were used for the enemy release experiment.

### 2.4 Enemy release experiment

We exposed native and invasive, inbred and outbred *S. latifolia* plants from the F2 generation to an enemy exclusion and an enemy inclusion treatment using a fully factorial experimental approach (16 populations [8 native and 8 invasive] × 4–5 families × 2 breeding treatments [inbred and outbred] × 2 enemy treatments [exclusion and inclusion] × 8 replicates = 1,224 plants). In early spring, we germinated eight seeds originating from one capsule per population × family × breeding treatment combination and reared the F2 plants for six weeks in a common garden in Halle (Saale), Germany (51.489°N 11.959°E alt: 88 m). After 6 weeks, we moved the plants to the UFZ Research Station in Bad Lauchstädt, Germany (51.391°N, 11.878°E, alt: 116 m). The planting area was densely covered by a diverse plant community of grasses and forbs including a patchy population of *S. latifolia* that was infested by all of the above‐mentioned specialist and generalist enemies. In the common garden, we established four vegetation‐free belts, which comprised four 5 × 6.5 m plots, respectively (∑ = 16 plots) (Supporting Information Figure S4). Each plot included all native and invasive populations represented by two to three maternal families each with one inbred and one outbred individual. As such, the five families within each population were split between two plots (plot pair), which together comprised all of the 153 population × family × breeding treatment combinations. Each plot pair was replicated an additional seven times. While populations and families were planted randomly within the plots, the range and breeding treatments were uniformly distributed according to a fixed scheme (Supporting Information Figure S4) in order to reduce confounding plot edge effects. Plots within pairs and plot pair repetitions were randomly distributed across the experimental area. We experimentally excluded natural enemies in eight of the plots (enemy exclusions) over a period of three months (Supporting Information Figure S4). For this purpose, we used slug fences coated with a gastropod deterrent (Schneckenabwehrpaste, Irka, Mietingen, GE), as well as a molluscicide (Limex, Celaflor), systemic insecticides (alternating between Calypso and Confidor, Bayer, Leverkusen, GE), and a systemic universal fungicide (Baycor M, Bayer, Leverkusen, GE), which were applied in a two‐week cycle in accordance with the manufacturers’ instructions. The remaining eight plots (enemy inclusions) were not treated with pesticides and therefore extensively colonized by specialist and generalist herbivores two weeks after the experiment was set up. The removal of vegetation, however, deterred *A. lusitanicus* from entering the inclusion plots, so we equipped them with slug fences whose impassable sides were turned toward the plot interior and introduced 15 *A. lusitanicus* individuals to each plot. This corresponds to the average number of slugs in four 5 × 6.5 m patches of undisturbed vegetation close to the experimental plots recorded at dusk on a humid day. We adjusted the number of slugs within each inclusion plot to 15 three times a week. The infection with specialist and generalist fungi remained low in all inclusion plots for the entire experimental period. All plots were weeded weekly and watered when necessary during the experiment.

After three months of exposure to or protection from natural enemies, we collected data on defense‐related traits in the enemy inclusion plots. We collected leaves at similar stages of development to determine trichome density in a 5 × 5 mm area away from the main vein and at the broadest section of the leaf. In addition, we determined the proportion of flowers (including buds) damaged by tissue removal (generalist herbivores) or phloem sucking (*B. lychnidis)*, the proportion of fruits predated by *H. bicruris* larvae, and the proportion of fully grown leaves infested by generalist herbivores (mainly *A. lusitanicus* and *M. brassicae*) in all experimental plots. These data confirmed the efficiency of the enemy exclusion/inclusion treatments (leaf, flower, and fruit damage in % ±SE: exclusions 1.52 ± 0.17, 0.14 ± 0.10, 0.14 ± 0.08; inclusions 28.79 ± 1.49, 9.78 ± 1.13, 5.66 ± 0.44). Data from inclusions were further used to analyze the effects of range and breeding treatment on defense‐related traits. Data on infection rates with the specialist fungus *M. violoceaum* and other generalist fungi were not included in these analyses, as the abundance of these pathogens was generally very low (only 0.12% and 0.37% of experimental plants infected). Furthermore, we collected data on plant growth and reproduction in both enemy inclusion and exclusion plots to address the interactive effects of range, breeding treatment, and herbivory treatment. We assessed the corolla diameter of the largest flower by identifying the 3–5 biggest flowers *via* visual inspection and measuring them. Moreover, we counted the number of flowers (including buds) for all male and female plants and determined the number of fruits for all female plants. Seed number and mass per plant were not assessed because many fruits were ripe and opened at the time point of data acquisition and thus already released seeds. Finally, we destructively harvested all plant individuals to determine their dry aboveground biomass (48 hr, 80°C). The experimental area was treated with herbicides in the following autumn and spring in order to prevent the establishment of experimental genotypes.

### 2.5 Statistical analysis

All statistical analyses were conducted with R version 3.4.1 (R Core Team, 2017). We used linear mixed‐effects models (LMMs) for response variables with Gaussian errors and generalized linear mixed‐effects models (GLMMs) for responses with Poisson or binomial errors (R package: lme4; Bates et al., 2014) with their default link functions.

The models for the defense‐related responses, trichome density (GLMM, Poisson, log‐link), leaf damage (GLMM, binomial, logit‐link), flower damage (GLMM, binomial, logit‐link), and fruit damage (GLMM, binomial, logit‐link) from enemy inclusion plots only, comprised the fixed effects of range and breeding treatment as well as an interaction among both factors. The models for the fitness‐related responses, biomass (LMM, Gaussian, identity‐link, square‐root transformed), corolla size (LMM, Gaussian, identity‐link), number of flowers (GLMM, Poisson, log‐link), and number of fruits (GLMM, Poisson, log‐link) from enemy exclusions and inclusions, comprised the fixed effects of range, breeding treatment, and enemy treatment as well as all possible interactions among these factors. All of the described models additionally involved the latitudinal coordinates of the population of origin (centered and scaled) and plant sex (except for fruit damage and number of fruits) as covariates. Moreover, all models included the random effects of plot, population, affiliation of paternal plant in P generation to field‐collected family nested within population, and affiliation of maternal plant in P generation to field‐collected family nested within population. Given the selected breeding scheme (see previous section), these random factors cannot fully account for the entire nonindependence arising from the individuals’ relatedness. However, we consider the above‐mentioned caveats that would have arisen from bias in reciprocal or independent pairings more severe.

All models were fitted with a maximum likelihood approach. We validated the chosen model types, link functions, and data transformations by assuring that all (G)LMMs exhibit variance homogeneity and normal distribution of residuals *via* visual inspection of model checking plots (Zuur, Ieno, Walker, Saviliev, & Smith, 2009). Moreover, GLMMs were tested for under‐ and overdispersion (R package: blemco, Korner‐Nievergelt et al., 2015). The GLMMs for leaf damage and number of flowers were overdispersed and consequently complemented by an observational‐level random factor in order to improve the model fit and avoid biased parameter estimates (Harrison, 2014, 2015). Following model checking, we applied stepwise backward selection to all models by removing fixed‐effect terms with *p* > 0.05 based on likelihood ratio tests (R package: MASS, Venables & Ripley, 2000). If breeding treatment was involved in a significant interaction in the minimal adequate model, we performed Tukey post hoc comparisons between inbreds and outbreds within both ranges (resistance‐related traits) or within all range × enemy treatment combinations (fitness‐related traits) (R package: lsmeans, Lenth, 2016). For illustration of the interactive effects of range, breeding treatment, and enemy treatment on plant performance responses, we extracted least square means with standard errors from the respective full mixed‐effects models (R package: lsmeans). In contrast to raw data means and their standard errors, these model estimates account for the specific error distribution of the responses, for the effects of covariates as well as for random effects.

To illustrate and discuss variation in I × E interaction effects among populations, we calculated the coefficient of inbreeding depression (δ) for all four fitness‐related traits (biomass, corolla diameter, number flowers, and number fruits) in enemy exclusions and inclusions on the population level as *(trait value outbred ‐ trait value inbred)/trait value outbred* (Keller & Waller, 2002) after standardizing all trait variables to the female parameter estimates (Table 1).

**Table 1:** Overview and results of analyses evaluating the interactive effects of range, breeding treatment, and enemy treatment as well as the effects of covariates (sex, latitudinal origin of population) on the performance of *Silene latifolia*

Response	Leaf trichomes	Leaf damage	Flower damage	Fruit damage	Biomass	Corolla diameter	Number flowers	Number fruits
**Model type** (error)	**GLMM (Poisson)**	**GLMM (binomial)**	**GLMM (binomial)**	**GLMM (binomial)**	**LMM (Gaussian)**	**LMM (Gaussian)**	**GLMM (Poisson)**	**GLMM (Poisson)**
**Model link function**	**Log**	**Logit**	**Logit**	**Logit**	**Identity**	**Identity**	**Log**	**Log**
Fixed effects	Estimate^sign.^	Estimate^sign.^	Estimate^sign.^	Estimate^sign.^	Estimate^sign.^	Estimate^sign.^	Estimate^sign.^	Estimate^sign.^
(Intercept)	4.269	−1.722	−2.932	−4.026	3.843	2.702	2.327	2.407
Range_(invasive)_	Dropped^n.s.^	0.574*	0.498**	**1.388^i.i.^**	**−0.008^i.i.^**	dropped^n.s.^	dropped^n.s.^	**0.421^i.i.^**
Breeding_(inbred)_	Dropped^n.s.^	0.645***	0.319***	**0.394^i.i.^**	−0.479***	−0.188***	−0.2***	**−0.161^i.i.^**
Enemy_(inclusion)_	‐	‐	‐	‐	**−0.14^i.i.^**	dropped^n.s.^	dropped^n.s.^	**−0.058^i.i.^**
Sex_(male)_	Dropped^n.s.^	Dropped^n.s.^	0.165*	‐	−0.314***	0.173***	2.095***	‐
Latitudinal origin population	Dropped^n.s.^	Dropped^n.s.^	Dropped^n.s.^	Dropped^n.s.^	Dropped^n.s.^	Dropped^n.s.^	Dropped^n.s.^	Dropped^n.s.^
Range:breeding	Dropped^n.s.^	Dropped^n.s.^	Dropped^n.s.^	−0.794*	Dropped^n.s.^	Dropped^n.s.^	Dropped^n.s.^	0.132*
Range:enemy	‐	‐	‐	‐	−0.188*	Dropped^n.s.^	Dropped^n.s.^	Dropped^n.s.^
Breeding:enemy	‐	‐	‐	‐	Dropped^n.s.^	Dropped^n.s.^	Dropped^n.s.^	−0.095*
Range:breeding:enemy	‐	‐	‐	‐	Dropped^n.s.^	Dropped^n.s.^	Dropped^n.s.^	Dropped^n.s.^
Random effects	Variance^(groups)^	Variance^(groups)^	Variance^(groups)^	Variance^(groups)^	Variance^(groups)^	Variance^(groups)^	Variance^(groups)^	Variance^(groups)^
Population:individual	n.t.^(551)^	1.222^(572)^	n.t.^(571)^	n.t.^(282)^	n.t.^(1192)^	n.t.^(1128)^	0.419^(1192)^	n.t.^(579)^
Population:father	0.013^(79)^	0.097^(79)^	0.248^(78)^	0.343^(75)^	0.049^(79)^	0.007^(79)^	0.004^(79)^	0.072^(79)^
Population:mother	0.017^(76)^	0.045^(76)^	0.24^(76)^	0.271^(73)^	0.109^(76)^	0.009^(76)^	0.005^(76)^	0.099^(76)^
Population	0.004^(16)^	0.139^(16)^	0.007^(16)^	0^(16)^	0.064^(16)^	0.007^(16)^	0.051^(16)^	0.017^(16)^
Plot	0.005^(8)^	0.148^(8)^	0.043^(8)^	0.127^(8)^	0.054^(8)^	0.002^(16)^	0.059^(16)^	0.071^(16)^
Residuals	n.e.^(551)^	n.e.^(572)^	n.e.^(571)^	n.e.^(282)^	0.544^(1192)^	0.176^(1128)^	n.e.^(1192)^	n.e.^(579)^

Note

Performance responses are presented with the applied model type, error distribution, and link function. The table presents parameter estimates for each of the fixed‐effect terms retained in the respective minimal adequate mixed‐effects model (main effects presented with second factor level in parenthesis) with their levels of significance (^n.s.^ *p* > 0.05, ^*^ *p* < 0.05, ^**^ *p* < 0.01, ^***^ *p* > 0.001) obtained from likelihood ratio tests. Significant effects are printed bold. Variance estimates for the random effects and residuals (main effects presented with the number of groups in parenthesis) are also provided.

GLMM: generalized linear mixed‐effects model; i.i.: fixed‐effect term in significant interaction; LMM: linear mixed‐effects model; sign: level of significance.

## 3 RESULTS

### 3.1 Interactive effects of range and breeding treatment on defense‐related traits

**Figure 1:**
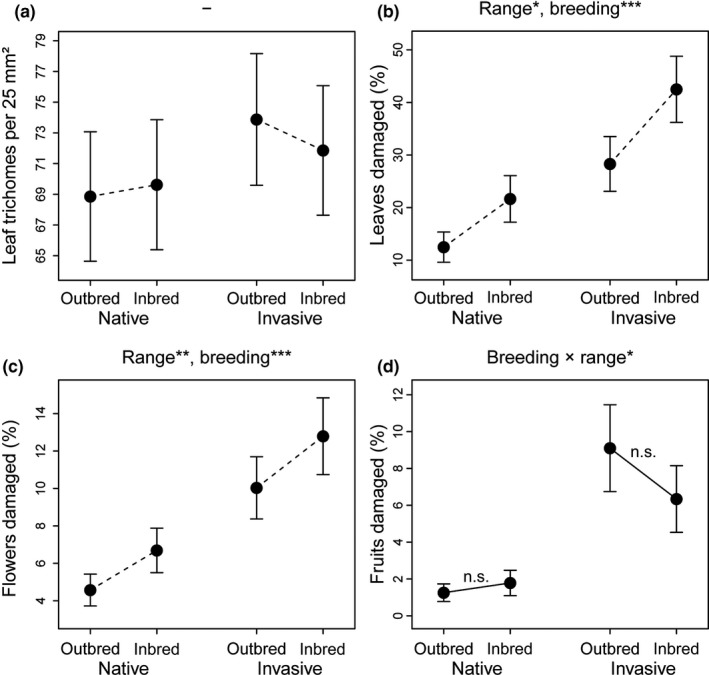
Combined effects of range (native vs. invasive) and breeding treatment (outbred vs. inbred) on defense‐related traits in *Silene latifolia* plants assessed in enemy inclusions. The significance levels for each effect (determined with likelihood ratio tests) are denoted at the top of each plot (n.s.: *p* > 0.05, *p < 0.05, **p < 0.01, ***p < 0.001). The circles represent least square means with standard errors from the full (G)LMMs. Dashed connection lines between means of inbreds and outbreds mark additive effects of breeding treatment with range, while solid lines highlight significant range × breeding treatment interactions. Tukey post hoc comparisons for breeding effects within each range were performed on models including a significant interaction, and their results (levels of significance) are denoted at the respective connection lines

The density of leaf trichomes was not significantly influenced by range, breeding treatment, the interaction range × breeding treatment, or one of the covariates (Table 1, Figure 1a). The proportion of damaged leaves was significantly related to range and breeding treatment (Table 1). Invasive plants experienced more leaf damage compared to native plants (*p* = 0.02, χ^2^ = 5.39), and inbred plants from both distribution ranges suffered stronger from leaf infestation compared to outbreds (*p* < 0.001, χ*^2^* = 41.69) (Figure 1b). The proportion of damaged flowers depended significantly on range, breeding treatment, and the covariate sex (Table 1). Flower infestation was higher for invasive than native (*p* = 0.01, χ^2^ = 6.79), inbred than outbred (*p* < 0.001, χ^2^ = 40.98) (Figure 1c), and male than female plants (*p* = 0.02, χ^2^ = 5.22). The proportion of damaged fruits was significantly influenced by the interaction range × breeding treatment (*p* = 0.04, χ^2^ = 4.12). Here, invasive plants received generally more fruit damage than native plants and fruit infestation was higher on inbred than outbred native plants but lower on inbred than outbred invasive plants (Figure 1d). Tukey post hoc comparisons among outbreds and inbreds within both ranges demonstrated that the inbreeding effect was not significant within the native (*p* = 0.64) and invasive range (*p* = 0.18).

### 3.2 Interactive effects of range, breeding treatment, and enemy treatment on fitness‐related traits

**Figure 2:**
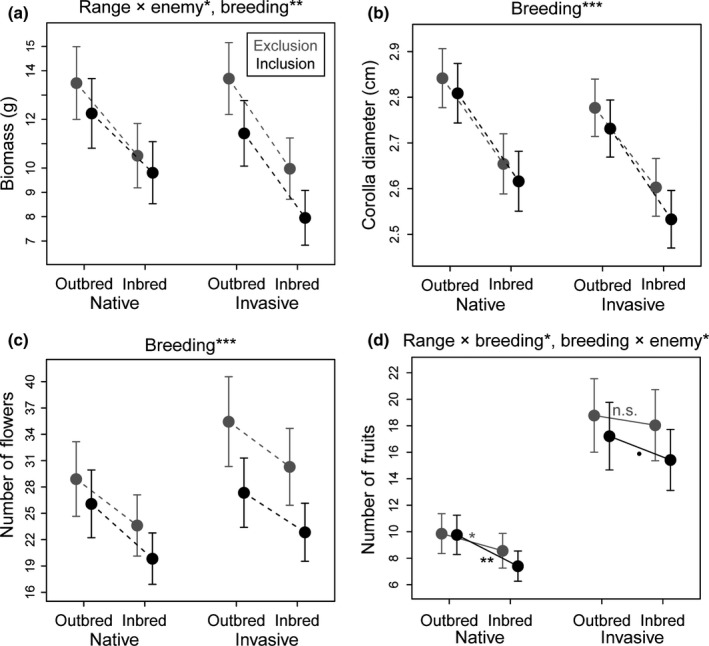
Combined effects of range (native vs. invasive), breeding treatment (outbred vs. inbred), and enemy treatment (exclusion [gray] vs. inclusion [black]) on fitness‐related traits in *Silene latifolia*. The significance levels for each effect (determined with likelihood ratio tests) are denoted at the top of each plot (n.s.: *p* > 0.05, ^•^ *p* < 0.06, *p < 0.05, **p < 0.01, ***p < 0.001). The circles represent least square means with standard errors from the full (G)LMMs. Dashed connection lines between means of inbreds and outbreds mark additive effects of breeding treatment with range or enemy treatment, while solid lines highlight significant interactions in which breeding treatment is involved. Tukey post hoc comparisons for breeding treatment effects within each range and enemy treatment were performed on models including such significant interactions, and their results (levels of significance) are denoted at the respective connection lines.

The aboveground biomass of experimental plants was significantly related to the interaction range × enemy treatment, to breeding treatment, and to plant sex (Table 1, Figure 2a). Plants exhibited reduced biomass in enemy inclusions relative to exclusions; whereby, this effect was stronger in invasive than native populations (*p* = 0.03, χ^2^ = 4.77). Inbred plants produced significantly less biomass compared to outbred plants (*p* = <0.001, χ^2^ = 116.63), and female plants had higher biomass than males (*p* ≤ 0.001, χ^2^ = 44.51). Range, breeding treatment, and enemy treatment had no significant interactive effects on the corolla diameter of *S. latifolia* plants (Table 1). Instead, corolla size was generally lower for inbred than outbred (*p* ≤ 0.001, χ^2^ = 54.46) (Figure 2b) and female than male plants (*p* ≤ 0.001, χ^2^ = 41.42). The number of flowers per plant individual was distinctively lower for inbred than outbred (*p* ≤ 0.001, χ^2^ = 24.50) (Figure 2c) and female than male plants (*p* ≤ 0.001, χ^2^ = 132.73). The number of fruits produced by female plants depended significantly on the two‐way interactions range × breeding treatment and breeding treatment × enemy treatment (Table 1, Figure 2d). Invasive plants produced more fruits than native plants in both breeding and enemy treatments. Moreover, inbred plants had fewer fruits than outbred plants and this inbreeding depression was less intense in invasive than native populations (*p* = 0.02, χ^2^ = 5.87) and stronger in enemy inclusions than exclusions (*p* = 0.04, χ^2^ = 4.15). Tukey post hoc comparisons among outbreds and inbreds within all four range × enemy treatment combinations further supported these model predictions: Inbreeding depression was generally significant in native populations and more pronounced in inclusions (*p* = 0.01) than exclusions (*p* = 0.04). In invasive populations, inbreeding depression was not significant in exclusions (*p* = 1.00), but became marginally significant in inclusions (*p* = 0.06).

### 3.3 Population variation in I × E interaction effects on fitness‐related traits

**Figure 3:**
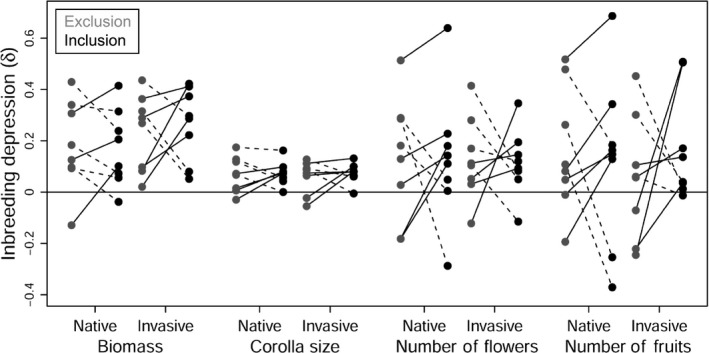
Population variation in I × E interaction effects on fitness‐related traits. Points represent the coefficient of inbreeding depression (δ) for biomass, corolla size, the number of flowers, and the number of fruits in eight native and eight invasive *Silene latifolia* populations exposed to an enemy exclusion (gray) and enemy inclusions (black) treatment. Lines connect δs for enemy exclusions and inclusions within each population. Solid lines highlight an increase in inbreeding depression under enemy attack, while dashed lines highlight reduced inbreeding depression under enemy attack

The magnitude of inbreeding depression (δ) exhibited pronounced variation across populations and traits (Figure 3). Variability in the coefficient of inbreeding depression was highest for fruit number (δ = −0.37 to 0.69) and flower number (δ = −0.28 to 0.63) and lowest for corolla diameter (δ = −0.05 to 0.17). Likewise, variable were the effects of enemy attack on the expression of inbreeding depression; whereby, δ increased in 56% of all population × trait combinations in enemy inclusions. The most severe increases in the magnitude of inbreeding depression under enemy attack were observed for the number of fruits in the two invasive populations ac (δ_exclusion_ = −0.07, δ_inclusion_ = 0.51) and es (δ_exclusion_ = −0.24, δ_inclusion _= 0.51).

## 4 DISCUSSION

### 4.1 Inbreeding increases infestation damage in native and invasive plant populations

In accordance with our hypothesis, inbred *S. latifolia* plants from both distribution ranges for the most part incurred higher infestation damage from natural enemies in the common garden than outbreds (Figure 1b,c, but see Figure 1d). Plants often exhibit increased susceptibility to enemies following inbreeding (Bello‐Bedoy & Núñez‐Farfán, 2011; Muola, Mutikainen, Laukkanen, Lilley, & Leimu, 2011; Stephenson, Leyshon, Travers, Hayes, & Winsor, 2004), since dominance and overdominance can either reduce the expression of genes that contribute directly to plant resistance against enemies (Kariyat, Mena‐Alí et al., 2012; Portman et al., 2015) or induce general stress responses that trade‐off against responses to environmental stressors such as natural enemies (Kristensen et al., 2010). Using the same inbred and outbred families of native and invasive *S. latifolia* populations investigated in the present study, Schrieber et al. (2018) demonstrated that inbreeding significantly compromises the plants’ metabolic responses to insect herbivory. This study also indicated that higher infestation damage on inbred *S. latifolia* individuals can result from compensatory feeding triggered by poor host plant quality. Previous studies on other plant species also demonstrated that inbreeding affects the composition and concentration of phenolic compounds mediating direct defense (Campbell et al., 2013) and volatiles mediating indirect defense (Kariyat, Mauck et al., 2013, 2012) as well as host plant quality (Leimu, Kloss, & Fischer, 2008).

In contrast to leaf and flower damage, fruit damage was significantly contrarily affected by inbreeding in native and invasive populations (Figure 1, Table 1). The proportion of fruits infested by *H. bicruris* was slightly higher in inbred than outbred native plants, but lower in inbreds than outbreds within invasive populations (Figure 1d). Although the breeding effects within each range were nonsignificant, this finding highlights that genetic differentiation and demographic disequilibrium can synergistically shape the attractiveness of *S. latifolia* to *H. bicruris,*which is a complex trait composed of flower morphology, number, and size as well as the composition of floral volatiles (Dötterl et al., 2006; Dötterl, Jürgens, Wolfe, & Biere, 2009; Elzinga & Bernasconi, 2009). The attractiveness of *S. latifolia* to herbivores was shaped not only by inbreeding but also by plant sex. Males received significantly more flower damage than females (Table 1), likely because their higher flower number (Table 1) attracted more specialist aphids and generalist chewing–biting herbivores.

### 4.2 Enemy release mitigates inbreeding depression in native and invasive plant populations

Both inbreeding and enemy infestation reduced the growth and reproduction of *S. latifolia*in native and invasive populations, whereby inbreeding had a pronounced effect and enemy infestation a rather weak effect (Figure 2). As hypothesized, the effects of breeding and enemy treatment were not purely additive. The magnitude of inbreeding depression was independent of the enemy treatment for biomass, corolla diameter, and flower number (Figure 2a,b,c), but significantly lower in enemy exclusions than inclusions for fruit number (Figure 2d).

While some studies found that herbivory increases inbreeding depression in multiple traits related to both growth and reproduction (Campbell et al., 2013; Carr & Eubanks, 2002), other studies also observed that I × E interactions only affect late live history traits very closely linked to reproductive success (Bello‐Bedoy & Núñez‐Farfán, 2011; Schou, Loeschcke, & Kristensen, 2015). The latter can occur, since the investment in reproduction by the end of a growing season is highly dependent on an individual's cumulative performance and thus on the cumulative effects of inbreeding and stress (natural enemies) on performance throughout the season (Orr, 2009). Our finding supports that the release from coevolved native enemies in the invaded habitat has the potential to mitigate detrimental inbreeding effects on reproductive output in plant populations experiencing demographic bottlenecks, which holds important implications for plant invasion success. The I × E interactions detected for native plants under experimental conditions may be representative of a scenario of initial population founding during early invasion phases, in which individuals are naïve to the novel environment. I × E interactions in the investigated invasive plants, in turn, may represent a scenario of population founding at the leading edge, where populations have already undergone evolutionary responses to the environment of the introduced range. If founder populations suffer less inbreeding depression in these crucial invasion phases, this might increase their establishment probability and eventually invasion success. However, the combined influence of breeding and enemy treatment observed in our study exhibited a high degree of variation across traits and populations (Figure 3) and is thus not generalizable. Moreover, the effect sizes for I × E interaction effects on fruit number in our study were low, and thus, it remains to be investigated whether they can indeed impact population growth rates. Future studies comparing estimates of inbreeding depression in plant invaders under benign and stressful conditions should thus ideally quantify seed output, viability, and germination as well as demographic rates in order to parameterize models that estimate population growth and spread rates (Normand, Zimmermann, Schurr, & Lischke, 2014; Schultz, Eckberg, Berg, Louda, & Miller, 2017).

### 4.3 Have I × E interactions contributed to invasion success in *S. latifolia*?

In contrast to our expectation, inbreeding effects on damage and I × E interaction effects on fitness were not more strongly pronounced in invasive than native populations, but rather equal in their magnitude in both ranges (Figures 1, 2). Hence, our results support that enemy release can mitigate inbreeding depression in *S. latifolia* populations, but not that this has indeed happened during the invasion of *S. latifolia*. If I × E interactions would have fostered the expansion of *S. latifolia* to North America, the relaxation of selection by natural enemies should have lead to the accumulation of deleterious recessive mutations in defense‐related traits and thus higher enemy‐induced inbreeding depression in invasive than native plants in our experiment (Schrieber & Lachmuth, 2017). The absence of these differences can be explained with two alternative evolutionary scenarios.

First, it is not only the relaxation from selection, but also low natural degrees of inbreeding in the history of a population that can lead to the accumulation of genetic load in specific traits (Leimu et al., 2008; Schrieber et al., 2017). Natural inbreeding exposes deleterious recessive mutations to negative selection. As a consequence, the frequency of these mutations within populations can rapidly decrease (i.e., purging of genetic load). If inbreeding levels are low, recessive mutations are masked in the heterozygous state, can be passed to the next generation, and thus accumulate in the population gene pool (Crnokrak & Barrett, 2002). Invasive *S. latifolia* populations have experienced increased inbreeding levels during colonization as evinced by inter‐ and intrapopulation crossing experiments (Richards, 2000), enhanced genetic structure in recently founded compared to longer established populations (McCauley, Raveill, & Antonovics, 1995), and the occurrence of severe demographic bottlenecks during initial founding (Keller et al., 2012; Taylor & Keller, 2007), whereas native, demographically more stable populations should have experienced comparably low levels of inbreeding (Austerlitz, Mariette, Machon, Gouyon, & Godelle, 2000). Consequently, genetic load in defense‐related traits may have accumulated in *S. latifolia* populations from both ranges based on different processes leading to equally pronounced I × E interaction effects: Native populations experienced selection by coevolved enemies, but low inbreeding rates/exposure of genetic load to selection; invasive populations experienced high degrees of inbreeding/exposure of genetic load to selection, but relaxation from selection by natural enemies. This evolutionary scenario is supported by the significantly lower inbreeding depression in fruit number in invasive relative to native populations (Figure 2d, Table 1). In contrast to defense‐related traits, reproductive traits are crucial for population fitness independently from enemy exposure and genetic load should thus experience strong negative selection if unmasked by inbreeding (Schrieber & Lachmuth, 2017). Our finding for range‐dependent inbreeding depression in fruit number thus suggests that invading populations experienced high natural inbreeding rates during colonization, which allowed purging for traits that are unconditionally crucial for population fitness (Burns, Ashman, Steets, Harmon‐Threatt, & Knight, 2011; Phillips, Brown, & Shine, 2010), whereas native populations did not. These results are consistent with those of a study on *Harmonia axyridis* Pallas (Facon et al., 2011) and further emphasize the importance of purging for the successful establishment and spread of invasive species.

Another possible explanation for equally pronounced I × E interaction effects in both distribution ranges is that invasive populations may have experienced selective pressure by generalist enemies native to North America, which counters the accumulation of deleterious recessive mutations in defense traits. This assumption is supported by the evolutionary differentiation in plant susceptibility to enemy infestation (Figure 1 b,c,d) and plant performance among native and invasive populations of *S. latifolia*(Figure 2d) detected in this study. Overall invasive plants received more infestation damage, but exhibited similar or even higher values for fitness‐related traits as native plants (Table 1). This observation has also been made in previous studies on *S. latifolia*(Blair & Wolfe, 2004; Schrieber et al., 2018, 2017; Wolfe et al., 2004) and suggests that invasive populations evolved increased tolerance of enemy infestation. The evolution of increased tolerance during range expansion has been observed in several other plant species (Abhilasha & Joshi, 2009; Zou, Rogers, & Siemann, 2007) and is assumed to arise from shifts in the natural enemy community, that is, reduced attack by specialists and increased attack by generalist (Fornoni, 2011).

Both of the alternative evolutionary scenarios outlined above are supported by our data, and they are mutually nonexclusive. This highlights that the combined effects of inbreeding and enemy infestation depend on the population history for selection by herbivores (differences in herbivore abundance and species composition) as well as the population history for inbreeding (frequency and intensity). Both of these factors can cause strong variation in inbreeding effects among distribution ranges (Figures 1d, 2d) and among populations within distribution ranges (Figure 3), and the exact direction of these effects should be addressed in future studies. These may combine records of infestation rates in field populations and highly resolved population genetic data (SNPs) with experimental stress applications in inbred and outbred populations from native and invasive origins. Moreover, a transplantation of inbred and outbred plants to native as well as invasive field habitats could assess the net effect of natural enemies and other environmental stressors (competitors, abiotic conditions) occurring in both environments on the magnitude of inbreeding depression. Studies of this kind could further elaborate whether and to what extent I × E interactions add to several other mechanisms (e.g., genetic admixture, mass introductions; Estoup et al., 2016; Rius & Darling, 2014) that can explain the successful spread of invaders in the face of genetic bottlenecks, that is, the so‐called genetic paradox of biological invasions.

## 5 CONCLUSIONS AND PERSPECTIVES

Our findings demonstrate that enemy release can mitigate inbreeding depression in plant populations. This supports the idea that I × E interactions have the potential to contribute to the successful establishment and expansion of introduced populations. On the other hand, I × E interactions might hamper the colonization of novel habitats that exhibit increased stress levels relative to a species’ native source habitat (Hufbauer et al., 2013; Rosche, Hensen, & Lachmuth, 2017). Furthermore, our data illustrate that the inbreeding effects on an organism's interaction with its environment are shaped by the evolutionary histories of populations. As the native and invaded range of a species can differ systematically in the stress regimes they experience, ongoing invasions provide ideal study systems for investigating the effects of evolutionary differentiation on the outcomes of I × E interactions, and how, in turn, the different outcomes may alter the evolutionary trajectories of invasive populations. Studies addressing these issues hold implications that extend far beyond invasive model species. I × E interactions may potentially shape the dynamics of natural populations whenever they are simultaneously exposed to habitat change and increased inbreeding rates following founder effects or population size reductions. These conditions occur not only during species range expansions, but also during range shifts and retractions in the course of global change (Colautti et al., 2017).

## AUTHOR CONTRIBUTIONS

SL and KS conceptualized the research and designed the study. KS conducted the field sampling, the experimental crossings, and the enemy release experiment and collected the data with assistance of CW, DH, and SW. KS analyzed the data. KS and SL interpreted the data. KS wrote the first version of the manuscript, and SL, SK, and IH contributed to the final version.

## Data Availability

All data that support this article have been deposited in Dryad (https://doi.org/10.5061/dryad.fb851dn).
