# Supplementary material for: Release from natural enemies mitigates inbreeding depression in native and invasive Silene latifolia populations
Source: Ecol Evol. 2019 Feb 18;9(6):3564–76. doi: 10.1002/ece3.4990 (PMC6434559; doi:10.1002/ece3.4990)

**Supporting Information Fig. S1**

**Fig. S1:** Map of the geographic locations of the sampled native (left) and invasive (right) *Silene latifolia* populations.


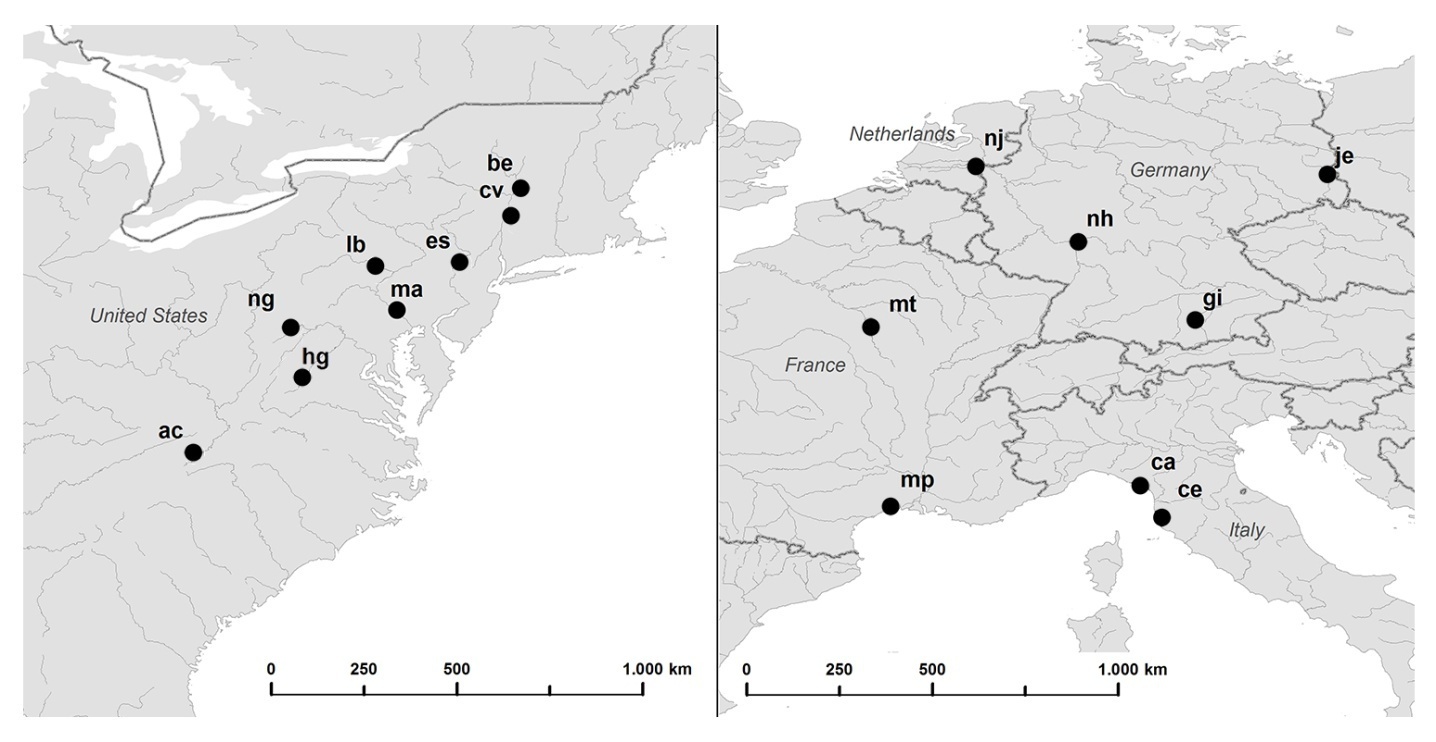

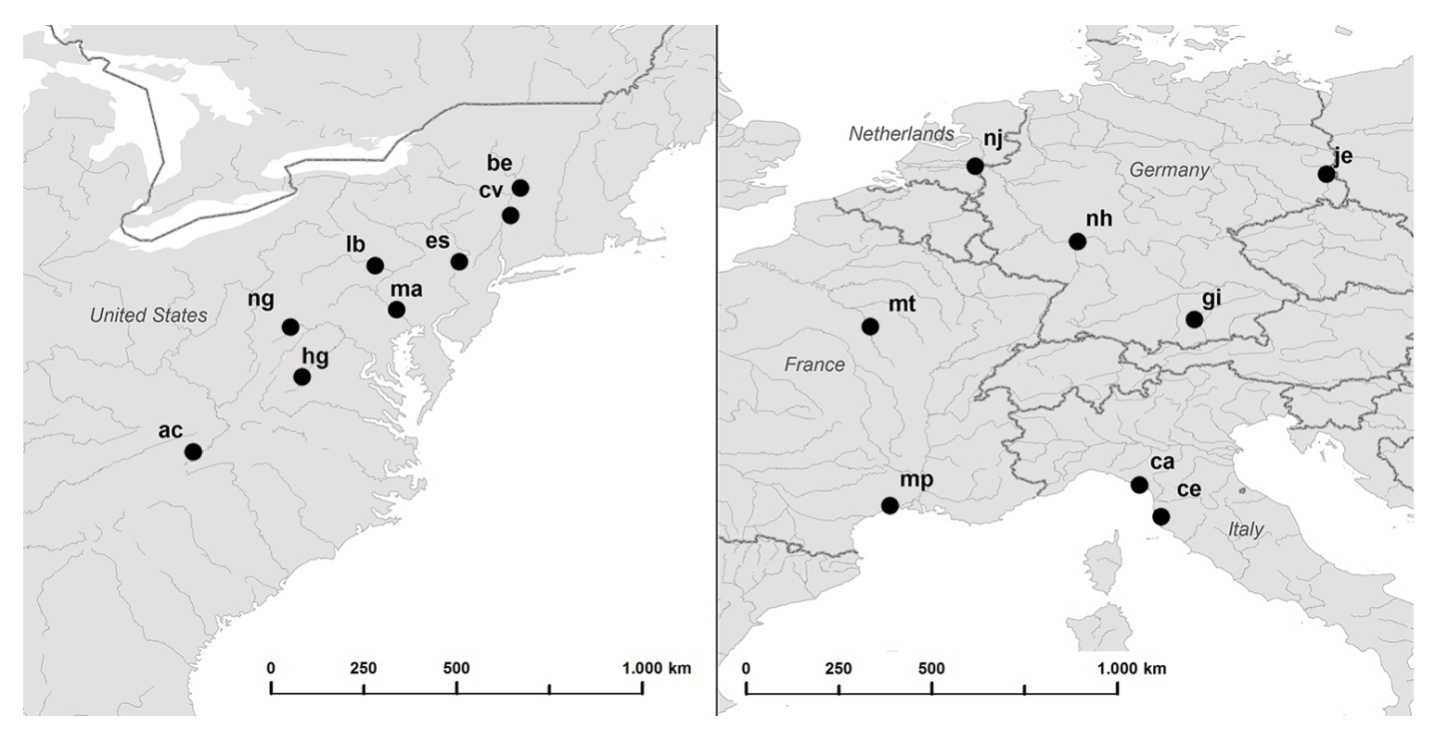

Supplement: Supplementary file 1 [file ECE3-9-3564-s001.docx]
