# Supplementary material for: Release from natural enemies mitigates inbreeding depression in native and invasive Silene latifolia populations
Source: Ecol Evol. 2019 Feb 18;9(6):3564–76. doi: 10.1002/ece3.4990 (PMC6434559; doi:10.1002/ece3.4990)
Supplement: Supplementary file 2 [file ECE3-9-3564-s002.docx]

**Supporting Information Table S2**

**Table S2:** Overview of the geographic locations and sizes of the sampled native and invasive *Silene latifolia* populations.

| Range | City (state) | ID | °N | °E | Population size |
| --- | --- | --- | --- | --- | --- |
| Native | Caresana (IT) | ca | 44.07702 | 9.96384 | 262 |
| Native | Cecina (IT) | ce | 43.313769 | 10.51195 | 149 |
| Native | Gilching  (GE) | gi | 48.105634 | 11.253771 | 35 |
| Native | Jethe (GE) | je | 51.6899 | 14.59683 | 485 |
| Native | Montpellier (FR) | mp | 43.653567 | 3.893247 | 341 |
| Native | Monteneau (FR) | mt | 47.848117 | 3.552508 | 19 |
| Native | Nackenheim (GE) | nh | 49.925119 | 8.342758 | 132 |
| Native | Nijmengen (GE) | nj | 51.883074 | 5.85185 | 46 |
| Invasive | Crumpler (NC) | ac | 36.52576 | -81.41558 | 60 |
| Invasive | Bennington (VT) | be | 42.894717 | -73.294919 | 31 |
| Invasive | Hillsdale (NY) | cv | 42.234825 | -73.506433 | 350 |
| Invasive | Bushkill (PA) | es | 41.14009 | -74.9294 | 900 |
| Invasive | Harrisonburg (VA) | hg | 38.49079 | -78.97762 | 70 |
| Invasive | Lewisburg (PA) | lb | 40.98179 | -76.93041 | 184 |
| Invasive | Washington Boro (PA) | ma | 39.99635 | -76.47243 | 1100 |
| Invasive | Grantsville (MD) | ng | 39.637308 | -79.099481 | 1000 |
