# Supplementary material for: Release from natural enemies mitigates inbreeding depression in native and invasive Silene latifolia populations
Source: Ecol Evol. 2019 Feb 18;9(6):3564–76. doi: 10.1002/ece3.4990 (PMC6434559; doi:10.1002/ece3.4990)

**Supporting Information Fig. S3**

**Fig. S3:** Overview of the two generations of experimental breeding within each of the 16 *Silene latifolia* populations. The crossings were performed with five field collected families (numbered circles). In the P-generation females (orange plants) were fertilized with pollen from males (green plants) from the same family for inbreeding (dashed arrows), and with pollen from males from a different family for outbreeding (solid arrows). In the P-generation, inbreeding and outbreeding were performed at distinct flowers of the same female individual. In the F1-generation, inbreeding was performed with individuals from inbred families and outbreeding with individuals from outbred families from the P-generation. Numbers for the F1-generation families correspond to the maternal/paternal plant of the breedings in the P-generation.


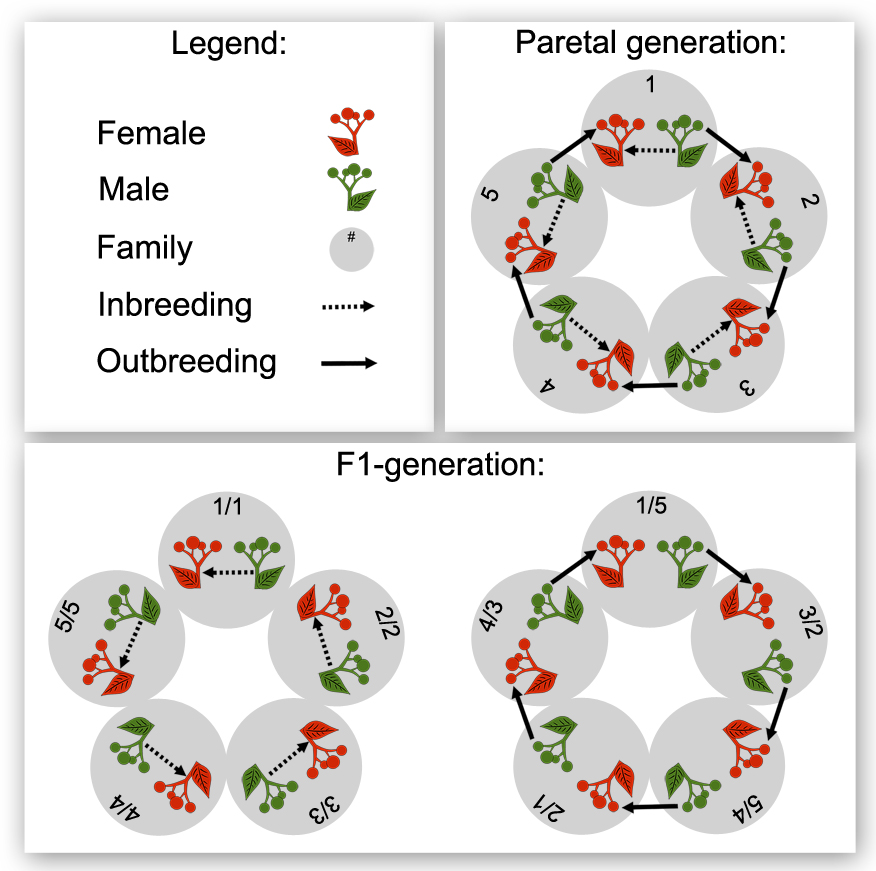

Supplement: Supplementary file 3 [file ECE3-9-3564-s003.docx]
