# Supplementary material for: Release from natural enemies mitigates inbreeding depression in native and invasive Silene latifolia populations
Source: Ecol Evol. 2019 Feb 18;9(6):3564–76. doi: 10.1002/ece3.4990 (PMC6434559; doi:10.1002/ece3.4990)

**Supporting Information Fig. S4**

## Fig. S4: Overview of the experimental manipulation of enemy infestation. The figure illustrates the non-vegetated areas (light gray faces) with the experimental plots (white faces) and the vegetated areas (structured, dark gray faces) from which natural enemies colonized the plots. Either the enemy exclusion (bold black frames) or the enemy inclusion (thin black frames) treatment was applied to each eight uniformly distributed plots. Within each plot, plants were equally distributed with respect to range (native = black, invasive = gray) and breeding treatment (filled = outcrossed, open = inbred).


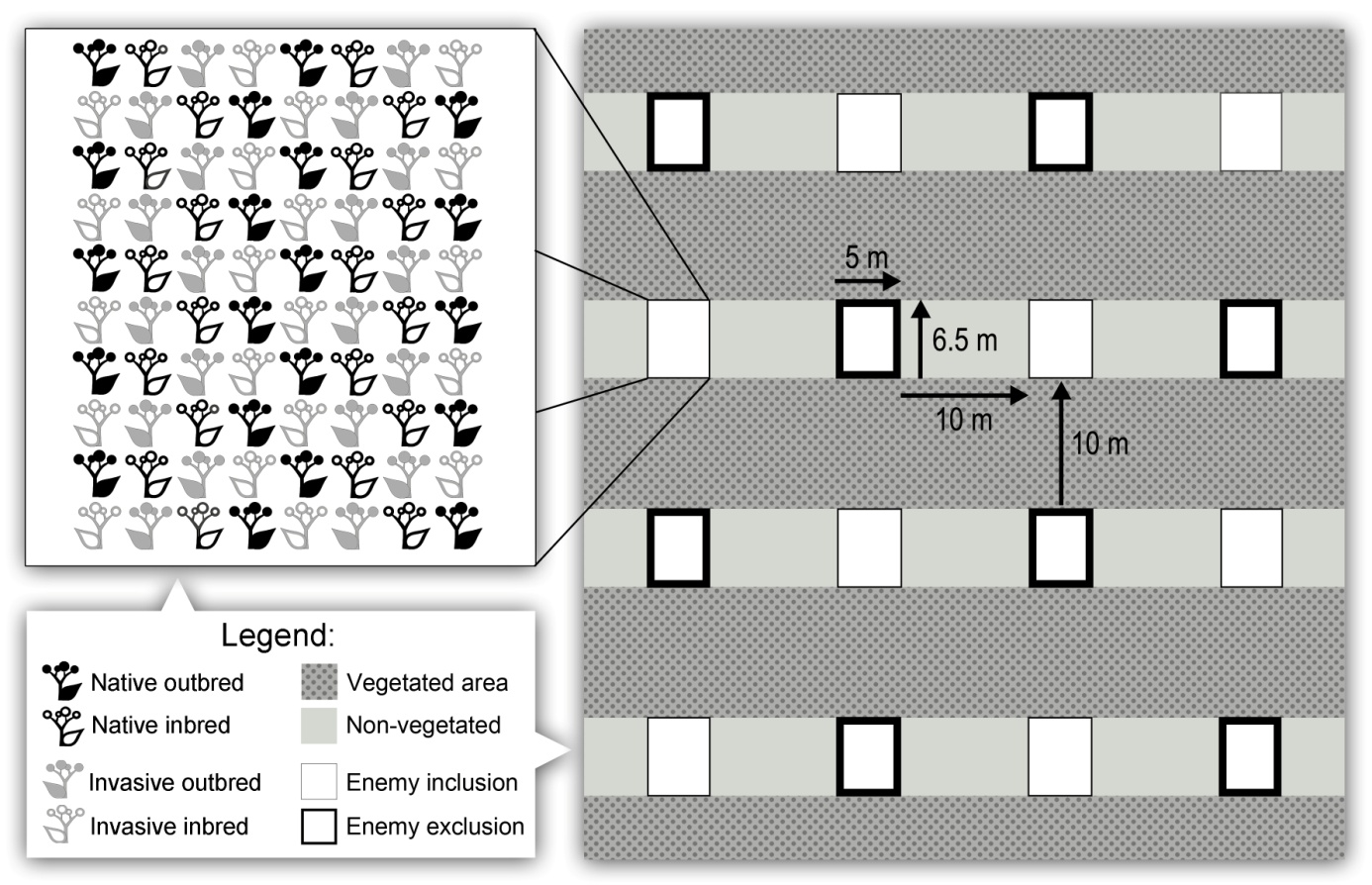

Supplement: Supplementary file 4 [file ECE3-9-3564-s004.docx]
